# The Institute of Medical Sciences

**Published:** 1905-04-01

**Authors:** 


### The Institute of Medical Sciences.

Mr. Alfred Beit has informed the honorary treasurers of the Institute of Medical Sciences Fund, University of London, that, having read the report of the committee appointed by King Edward's Hospital Fund on the financial relations of the hospitals and medical schools and the correspondence and articles thereon in the *Times*, he has decided to increase the amount of his donation to the Institute from £5,000 to £25,000. Mr. Beit desires by this gift to commemorate the great kindness of his friend, Dr. Jameson, C.B., the Premier of Cape Colony, and the services of members of the Faculty of Medicine during an illness from which he has now happily recovered. In such complimentary words Mr. Beit has made known the gift which he has given to the University of London at a critical time and for a most useful purpose. Mr. Beit's generosity will bring the establishment of an institute for the teaching of preliminary medical subjects within the range of practicability on the financial side, but much has to be done in the way of adjustment and levelling before even the foundations of a scheme could be brought forward which would have even the smallest chance of acceptance by the various medical schools in London. The Royal College of Surgeons have found it impracticable to open such a centre. The ideal scheme is, therefore, to concentrate the teaching of all the sciences ancillary to medicine including chemistry, physics, botany, biology, anatomy, and physiology at the University of London. A student would remain in this teaching centre at the University until he had passed in all these subjects, and he would then select the hospital at which he chose to carry out his purely professional training in pathology and in the medical and surgical wards. Difficulties will occur at the very beginning of the scheme from fears that there will be no longer an even distribution of clinical students amongst the schools. These fears are likely to prove groundless, for the influences which govern the choice of a school will be the same as those now in force. Good teaching, good material from which to teach, the influence of friends, the ties of heredity, cause students to enter at one hospital rather than another. Such influences must continue, and, in so far as teaching and clinical material are concerned, necessarily act for good upon the whole profession of medicine. A smaller school, in at least one instance, might lay itself out to become purely an honour school, selecting the cases in its wards, supplied from a large out-patient department, chiefly for their difficulty or for their points of interest. Such a school might admit students of proved ability reaching out for the higher examinations, and might dispense entirely with passmen, just as in various colleges at Oxford men are only admitted after an entrance examination of sufficient severity to show that they are capable of instruction in an honour school. Mere selfish and less academic motives are liable to hinder, if they do not entirely prevent, the formation of an institute for the teaching of the preliminary medical sciences. Each hospital has its own laboratories and staff for the teaching of these sciences, and in some cases these laboratories are specifically endowed, and could not well be abandoned. The staffs of the rest could well be amalgamated by the exercise of a little altruism, and the laboratories could be utilised for the extension of the pathological investigations which are now essential to the scientific practice of medicine and surgery. It would be inexpedient, therefore, to give up pathological work in favour of a central pathological institute, for questions of difficulty and importance are constantly arising which require answers from a pathologist of experience rather than from an advanced student or a member of the staff who used to be a pathologist. The present organisation of the Faculty of Medicine of the University of London does not make for the easy progress of a new scheme. But, in spite of all difficulties, several steps have been made in the direction of establishing an institute of medical sciences for the students of London. The University is established, the medical schools of the metropolis are affiliated to it, money has been obtained. The desire to amalgamate and the acquisition of suitable premises are all that is now needed. We trust that these may come speedily.

